# The emerging role of Slit-Robo pathway in gastric and other gastro intestinal cancers

**DOI:** 10.1186/s12885-015-1984-4

**Published:** 2015-12-16

**Authors:** Tingting Huang, Wei Kang, Alfred S. L. Cheng, Jun Yu, Ka Fai To

**Affiliations:** Department of Anatomical and Cellular Pathology, State Key Laboratory of Oncology in South China, Prince of Wales Hospital, The Chinese University of Hong Kong, Hong Kong, SAR PR China; Institute of Digestive Disease, Partner State Key Laboratory of Digestive Disease, The Chinese University of Hong Kong, Hong Kong, SAR PR China; Li Ka Shing Institute of Health Science, Sir Y.K. Pao Cancer Center, The Chinese University of Hong Kong, Hong Kong, SAR PR China; Shenzhen Research Institute, The Chinese University of Hong Kong, Shenzhen, PR China; School of Biomedical Sciences, The Chinese University of Hong Kong, Hong Kong, PR China; Department of Medicine and Therapeutics, The Chinese University of Hong Kong, Hong Kong, PR China

**Keywords:** Gastric cancer, Gastrointestinal cancers, SLIT, ROBO, Slit-Robo pathway, Oncogene, Tumor suppressor

## Abstract

Gastric cancer remains one of the most common cancers worldwide and one of the leading cause for cancer-related deaths. Due to the high frequency of metastasis, it is still one of the most lethal malignancies in which kinds of signaling pathways are involved in. The Roundabout (ROBO) receptors and their secreted SLIT glycoprotein ligands, which were originally identified as important axon guidance molecules, have implication in the regulation of neurons and glia, leukocytes, and endothelial cells migration. Recent researches also put high emphasis on the important roles of the Slit-Robo pathway in tumorigenesis, cancer progression and metastasis. Herein we provide a comprehensive review on the role of these molecules and their associated signaling pathway in gastric and other gastrointestinal cancers. Improved knowledge of the Slit-Robo signaling pathway in gastric carcinoma will be useful for deep understanding the mechanisms of tumor development and identifying ideal targets of anticancer therapy in gastric carcinoma.

## Background

Gastric cancer (GC) is the fourth-most-common cancer globally and the second-leading cause of the cancer deaths [1]. The potential risk factors include *Helicobacter pylori* (*H. pylori*) and EBV infection, high-salt and low-vegetable diet, smoking, chronic gastritis with glandular atrophy and intestinal metaplasia, and the most important factor is genetic alterations [2]. Currently, the main treatment option is the gastrectomy combined with chemotherapy and radiation therapy protocols. Unfortunately, survival of patients with advanced GC treated with palliative chemotherapy remains low. The poor prognosis associated with GC is mainly related with high frequency of metastasis. Tumor cell motility and invasion play fundamental roles in cancer metastasis. Therefore, an in-depth understanding of molecular bases of the GC is required.

GC is proposed to result from the complex interplay between genetic and environmental factors at the gastric mucosa level that deregulates cell potentially oncogenic signaling pathways, leading to GC development [3, 4]. Previous studies in GC revealed multiple oncogenic signaling pathways such as Wnt/β-catenin, nuclear factor-κB, Hedgehog, Notch and epidermal growth factor receptor are implicated in gastric carcinogenesis [5]. Activation of these signaling cascades leads to acquisition of malignant phenotypes including increased cell proliferation, evasion of apoptosis and enhanced invasiveness. Improved knowledge of these signaling pathways will help to understand the molecular mechanisms in GC and identify novel targets for anticancer drug development. In this brief review, the involvement of the Slit-Robo signaling pathway and its biological significance in gastrointestinal cancers will be summarized.

## The main components of Slit-Robo pathway

The Roundabout (Robo) gene encodes a transmembrane receptor that was firstly identified in Drosophila [6]. Six years later, the Drosophila SLIT protein was identified as the ligand for ROBO receptor [7]. SLIT proteins are secreted glycoproteins that mediate their functions by binding to the ROBO. There are three ROBOs (ROBO1, ROBO2 and ROBO3) and a single SLIT in Drosophila [8]. Thereafter, three SLITs (SLIT1, SLIT2, SLIT3) and four different ROBOs (ROBO1/DUTT1, ROBO2, ROBO3/RIG-1, ROBO4/Magic Roundabout) members have been described in human [9, 10].

SLIT1–3 are expressed in the nervous system and in most organs [11]. The most commonly studied member is SLIT2. It is known to regulate many aspects of tissue morphogenesis and cell function, including cell migration, proliferation, adhesion and death [12]. Although the genes overlap, their expression patterns and functions are distinct. SLIT1 is specific to the brain, and SLIT2 and SLIT3 are expressed in the brain as well as other tissues [13]. SLITs are characterized by four distinct domains. There are 4 leucine-rich repeats (LRR) domains at the N-terminus, termed D1-D4. This region is followed by six EGF-like sequences (EGF), a laminin-G domain and a C terminus with a cysteine-rich knot [14, 15]. Detailed structure-function studies have shown that the second LRR domain plays an important role in binding to ROBO proteins [16].

ROBO is a single-pass, transmembrane receptor belonging to the immunoglobulin superfamily. In mammals, ROBO1-3 is expressed in many tissues during development and particularly in the nervous system [17–19]. The latest member of this family ROBO4, also called magic roundabout, was originally thought to be expressed mainly by endothelial cells [20, 21]. ROBO1 and ROBO4 are two receptors of the secretory protein SLIT2. All ROBOs, with the exception of ROBO4, share a common extracellular domain structure of five immunoglobulin-like (Ig) motifs followed by three fibronectin type 3 (FNIII) domains and four conserved cytoplasmic (CC) domains, expressed in different combinations within the ROBO receptor family [22]. The cytosolic ROBO domains are poorly conserved, with the notable exception of several conserved linear motifs, CC0-CC3, which occur in different combinations in different ROBOs. The intracellular domains of ROBO1 and ROBO2 share the same conserved cytoplasmic motifs (CC0, CC1, CC2 and CC3). The CC1 motif is absent in ROBO3. ROBO4, which has the lowest homology with other ROBO family members, contains only two IG and FNIII domains along with one CC motif, CC2 [23]. The IG1 and IG2 of ROBO is essential for the Slit–Robo interaction [24].

Biochemical studies show that the interaction between SLIT and ROBO involves the highly conserved second LRR domain (D2) of SLIT and the IG1 domain of ROBO, while IG2–IG5 and all FN3 domains of ROBO1 appear to be dispensable for binding [16, 23, 25, 26]. Substantial evidence has been accumulated that Slit-Robo signaling strictly requires heparin sulfate (HS) proteoglycans [27, 28]. HS proteoglycans have been shown to stabilize the relatively weak Slit-Robo interaction [29].

## The physiological function of Slit-Robo pathway

Slit-Robo signaling was first established as an extracellular signature to guide axon path finding, promote axon branching and control neuronal migration. The interaction of SLIT and ROBO proteins is crucially involved in the development processes of various vital organs such as breast, lung, liver, kidney, eye and reproductive systems [24]. Function of Slit-Robo signaling is influenced by binding of intracellular factors to the cytoplasmic domains of ROBO. The cytosolic domains of ROBOs are catalytically inactive. ROBO activation at the cell surface is then translated within the cell by a number of signaling cascade events. Although it is best known as a conserved repellent cue for axon guidance during the development of the central nervous system, its other functions are becoming increasingly studied. This section will introduce the various physiological functions of Slit-Robo signaling.

### The role of Slit-Robo in nervous system development

The central nervous system (CNS) develops along a bilateral axis of symmetry located at the midline [30]. During development, the ventral midline or floor plate acts as an organizer through the secretion of diffusible proteins, which control the growth of axons and dendrites and the migration of neurons across the midline [30, 31]. Slit-Robo signaling was first established as extracellular signature axon guidance [8, 32]. They function as a repulsive cue with an evolutionarily conserved role in preventing axons from migrating to inappropriate locations during the assembly of the nervous system [8]. Furthermore, the Slit-Robo interaction has a similar function during the development of other processes in the nervous system including formation of the olfactory tract, optic chiasm, optic tract, forebrain and hindbrain [33].

### The role of Slit-Robo in cell motility

Slit-Robo signaling has been shown to control the migration of several neuronal subtypes in mice including cortical interneurons [34, 35], cerebellar granule neurons [36, 37] and inferior olivary neurons [38]. At the subcellular level, novel data suggest that SLITs control neuronal migration by influencing cell polarity. Calcium (Ca^2+^) signaling and Rho GTPases are known to modulate the radial migration of cortical neurons and cerebellar granule cells [39, 40]. In order to direct changes in cell motility, the binding of SLIT to the ROBO receptor leads to reorganization of the actin cytoskeleton. The binding of the SLITs modified the cytoplasmic domains of the ROBO receptors. Actin polymerization is regulated by several adaptor proteins that can bind to the cytoplasmic motifs of the ROBO receptors. In drosophila, the two proteins Ableson tyrosine and Enabled (Ena) mediate cytoskeletal remodeling downstream of Slit-Robo signaling [41]. In mammals, small GTPases of the Rho family, such as RhoA, Rac1 and Cdc42, are key regulators of actin cytoskeletal dynamics [42]. These proteins switch from an inactive GDP-bound state to an active GTP-bound state and are regulated by the GTPase-activating proteins (GAPs) and the guanine nucleotide-exchange factors (GEFs). GEFs activate Rho GTPases while GAPs inactivate them by inducing GTP hydrolysis. The binding of the ligand modifies the cytoplasmic domains of the ROBO receptors, which leading to the recruitment to the intracellular domain of ROBO1 interacts with Slit-Robo GAP1 (srGAP1) and leads to the inactivation of Cdc42 and a modest activation of RhoA and Rac [43, 44]. GTP-bound RhoA promotes the formation of actin stress fibers and focal adhesions and mediated cell repulsion. However, GTP-bound Cdc42 and Rac lead to cell attraction and promote the formation of filopodia and lamellipodia, respectively [45]. Thus, Slit2-Robo signaling dynamically regulates rearrangements of the actin cytoskeleton and influences multiple cellular processes [46].

### The role of Slit-Robo in angiogenesis

Slit-Robo signaling also has important roles in regulating both non-pathological and pathological angiogenesis. SLIT3, ROBO1 and ROBO4 are expressed by endothelial cells. ROBO4 in particular is exclusively expressed by endothelial cells [21, 47]. Indeed, morpholino-mediated knock down of ROBO4 leads to asynchronous intersomitic vessel sprouting, resulting in a reduction and misdirection of intersomitic vessels [48]. Recently identified ROBO4 is the key mediator of Slit-Robo mediated developmental and pathological angiogenesis. ROBO4 is expressed specially in vascular endothelial cells and maintains the vascular integrity via either SLIT2 dependent or SLIT2 independent manner. On the contrary, it promotes the pathological angiogenesis by involving different signaling arm(s)/unknown ligand(s) [49].

### Other physiological roles of Slit-Robo pathway

Slit-Robo signaling can regulate other cellular processes involved in cell growth. It can inhibit hepatocyte growth factor (HGF), stromal derived factor-1 (SDF-1) and β-catenin activity [50–52]. Furthermore, Slit-Robo signaling axis is also extensively involved in myogenesis [53], kidney induction [54], heart tube formation [55], leukocyte migration [13], periodontitis [56], and vascular injury [56].

## The deregulated Slit-Robo pathway in gastrointestinal cancers

Apart from physiological functions, Slit-Robo interactions are involved in many pathological cellular processes, including cell cycle, apoptosis, cell adhesion, motility, angiogenesis and invasion, which are important for tumorigenesis [57]. There is evidence that loss of ROBO1 in mice induces tumorigenic. Most ROBO1-knockout mice exhibit postnatal morbidity, but surviving mice suffer from bronchial hyperplasia and focal dysplasia [58]. The current data indicate that Slit-Robo pathways differentially modulate invasion and migration, which varies according to signaling and type of cancers. The first link between Slit-Robo signaling and the cancer was reported by Sundaresan [59].

Although the involvement of Slit-Robo signaling in tumor development has been widely implicated, the biological significance and molecular mechanisms of Slit-Robo signaling in the initiation of gastrointestinal cancers are largely undetermined. Gastrointestinal cancers refer to malignant conditions of the gastrointestinal tract and accessory organs of digestion, including the esophagus, stomach, biliary system, pancreas, small intestine, large intestine, rectum and anus.

### Gastric adenocarcinoma

The activation or suppression of the Slit-Robo pathway modulates several oncogenic signaling pathways that are associated with the development and progression of cancer [60, 61]. In 2003, Wang et al. demonstrated the angiogenesis promoting function of Slit-Robo signaling and evaluated the significance of this pathway in the pathogenesis of cancers. They examined human samples of different carcinomas and found in gastric squamous carcinoma samples, SLIT2 was expressed in the cancerous tissues, but not in the nearby regions of apparently normal tissues. The positive staining of SLIT2 was found in 38 gastric carcinomas. Their results have showed SLIT2 can attract vascular endothelial cells and promote tumor-induced angiogenesis. Furthermore, they demonstrated the neutralization of ROBO1 reduced the microvessel density and the tumor mass of human malignant melanoma A375 cells *in vivo* [3, 62].

Meanwhile, Wang et al. revealed that the NF-κB/POU2F2/SLIT2/ROBO1 network might play an essential role in GC metastasis. POU2F2 promoted GC metastasis by a positive regulation of ROBO1. The interaction between NF-κB and the SLIT2/ROBO1 pathway linked by POU2F2 contributed to GC metastasis [63].

Tie et al. have shown that GC metastasis is associated with the downregulation of a specific set of miRNAs, including miR-218-1, a miRNA hosted in an intron of the SLIT3 gene, which directly inhibits ROBO1 expression using both *in vitro* and *in vivo* approaches. They present a model in which the acquisition of metastatic propensity occurs as a result of the downregulation of miR-218-1 and an upregulation of ROBO1 [64]. Furthermore, miR-218 may also regulate ROBO1 function during angiogenesis [65].

Recent data from our laboratory showed a potential co-regulation of srGAP1 (Slit-Robo GTPase activating protein1) in GC (data not shown here). srGAP1 is a downstream component of Slit-Robo signaling. Secretory glycoprotein SLIT2 interacts with its membrane receptor ROBO1 leading to the activation of srGAP1. Currently it is unclear whether these genes are differentially regulated based on tumor type or stage, but mounting evidence suggests that changes in the expression of these genes play important roles in regulating tumor progression. We also identified ROBO1 was significantly up-regulated in some GC cell line when compared to normal gastric epithelium cells.

On the contrary in 2013, Zhang et al. described the expression pattern of SLIT2 in GC. Immunohistochemistry (IHC) staining revealed that SLIT2 was found decreasingly expressed in advanced-stage GC tissues when compared with early-stage GC [66]. Subsequently, they investigated the roles of SLIT2 in GC. They generated the SLIT2-knockdown gastric cell model and found SLIT2 knockdown promoted GC cell growth and metastasis *in vitro* and *in vivo*. SLIT2 knockdown increased GC cell growth in monolayer and soft agar/Matrigel 3D culture. SLIT2-knockdown cells formed larger tumors and produced more peritoneal metastatic nodules in nude mice. Subsequently, they suggested that knockdown of SLIT2 activates AKT, enhances anti-apoptotic ability and activates β-catenin oncogenic signaling. They suggested a tumor-suppressor role of SLIT2 in GC [67].

In addition, a research group found ROBO1 and ROBO2 frameshift mutations in both GC and colorectal cancer (CRC). Frameshift mutations of ROBO1 and ROBO2 genes and loss of ROBO1/2 expression in GC and CRC suggested that both genes might play roles in the pathogenesis of GC and CRC [68]. Meanwhile, ROBO1 or ROBO2 expression is frequently lost in many cancers, including head/neck, breast, lung, kidney and uterine cervix cancers, and is associated with methylation and loss of heterozygosity of these genes [69–71]. These data suggest that both ROBO1 and ROBO2 are potential tumor suppressor genes.

The Cancer Genome Atlas (TCGA) project described a comprehensive molecular evaluation of primary gastric adenocarcinomas [72]. According to the cBioPortal for Cancer Genomics which has comprehensively summarized the TCGA data, the SLIT2, ROBO1 and srGAP1 gene show 19 % (47/258), 13 % (31/258) and 12 % (29/258) cases with alterations. The alterations include somatic mutations, copy number changes (gene amplification or deletion) and mRNA expression change [73, 74].

**Fig. 1:**
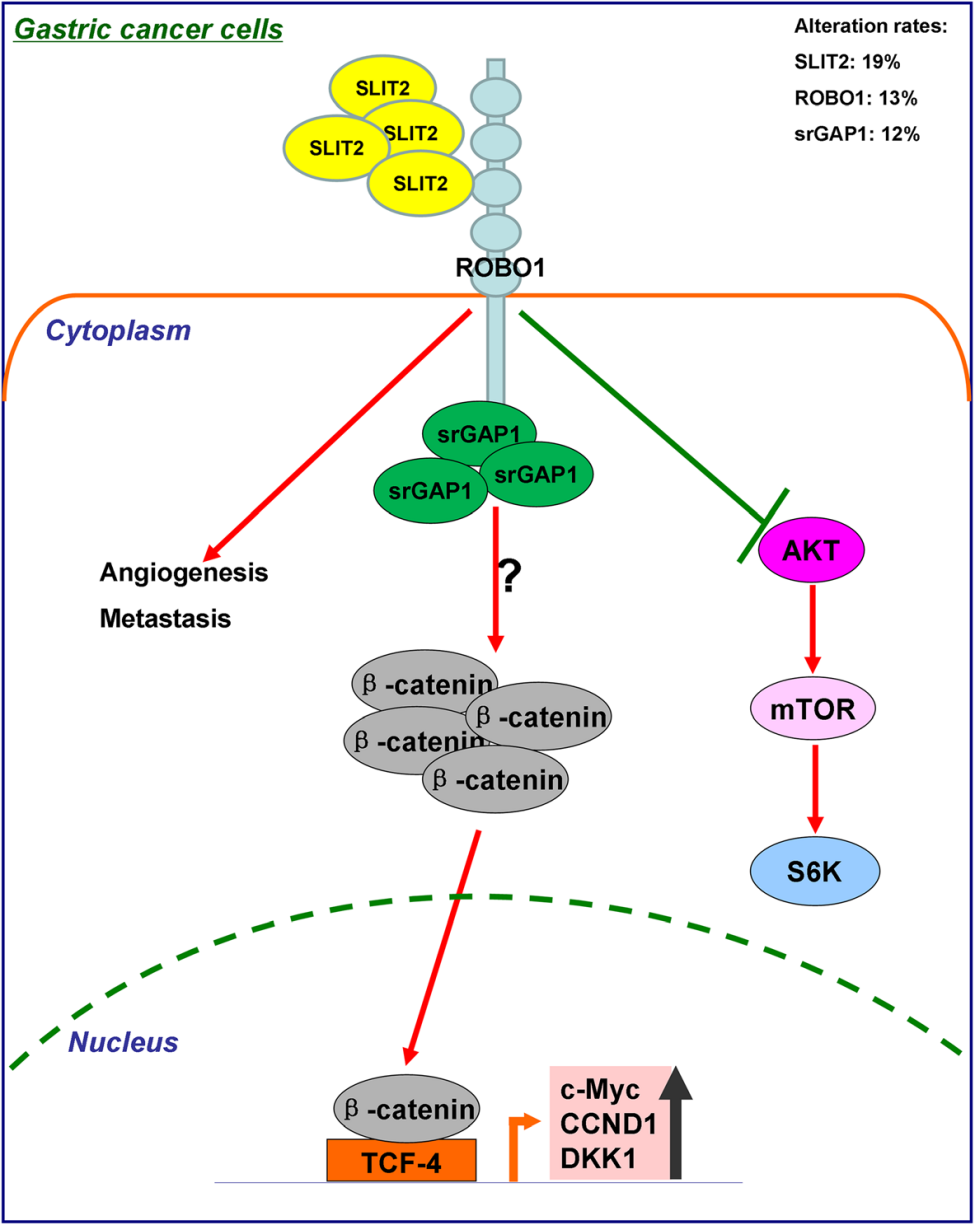
The schematic representation of Slit-Robo signaling pathway in gastric tumorigenesisᅟ

Overall, the functional study of Slit-Robo pathway in GC seems very contradictory by different research groups and further studies will be performed to elucidate the complex signaling pathway in gastric tumorigenesis (Fig. 1).

### Hepatocellular carcinoma (HCC)

Avci et al. quantified Slit-Robo transcripts in HCC cell lines, normal and tumor tissues from liver, and suggested that the expression of Slit-Robo genes were regulated in hepatocarcinogenesis. Slit-Robo expression predicted AFP-dependent subgrouping of HCC cell lines and ROBO1 was found to be significantly overexpressed in HCC [75]. Ito et al. identified of ROBO1 as a novel hepatocellular carcinoma antigen and a potential therapeutic and diagnostic target. They found ROBO1 was highly expressed in HCC, whereas it showed only a limited distribution in normal tissues. Strikingly, the ectodomain of ROBO1 was detected in sera from HCC patients [76]. Subsequently, a novel function for SLIT and ROBO in the inhibition of growth factor-mediated epithelial cell motility and morphogenesis has been disclosed. The Slit-Robo interaction inhibits hepatocyte growth factor (HGF)/MET-induced tumor cell migration and invasion [52].

### Colon cancer

Although efforts have been made to elucidate the role of Slit-Robo signaling in CRC, the results are also contradictory [77–79]. Zhou et al. found that the N-terminal domain of SLIT2 could induce malignant transformation of colorectal epithelial cell and tumor metastasis. They found that engagement of ROBO1 by SLIT2 recruits Hakai to E-cadherin, culminating in E-cadherin ubiquitination and lysosomal degradation [78]. Previously, they have reported elevated expression of SLIT2 in human colorectal carcinoma tissues and cell lines [62]. Using cDNA microarray, a significant upregulation of ROBO1 was found in colorectal carcinoma tissues [80]. Furthermore, knockdown of endogenous ROBO1 or specific blockade of SLIT2 binding to ROBO1 prevented E-cadherin degradation and reversed EMT (epithelium mesenchymal transition), resulting in diminished tumor growth and liver metastasis. In colorectal carcinoma patients, the overexpression of SLIT2 and ROBO1 was significantly associated with an increased metastatic risk and poorer overall survival in colorectal carcinoma patients.

On the contrary, Dallol et al. reported that SLIT2 is an excellent candidate tumor suppressor gene in CRC and frequently inactivated and suppresses the growth of CRC cells [77]. Yu et al. found the expression levels of miR-218, SLIT2 and SLIT3 in CRC tissues were decreased and miR-218 expression was significantly associated with TNM stage, lymph node metastasis and differentiation [81]. Subsequently, Chen et al. found SLIT2 suppresses colon tumor metastasis, and exerts suppressive activity against CRC metastasis by restraining AKT signaling and EMT [79]. Recently, Huang et al. found that the USP33 mediates Slit-Robo signaling in inhibiting CRC cell migration [82].

### Esophageal cancer

In esophageal cancers, SLIT2 is a migration suppressor for ESCC (esophageal squamous cell carcinoma) via inhibition of srGAP–Cdc42 signaling and membrane localization of p-FAK and p-Paxillin. Tseng et al. found that 31.8 % (49 of 54) of tumors from ESCC patients showed low expression of SLIT2 protein, which correlated with poor overall survival and disease-free survival. They also demonstrated that promoter hypermethylation of SLIT2 is responsible for low expression level in ESCC. SLIT2 overexpression decreased Cdc42 activity in ESCC cells, while knockdown of SLIT2 facilitated the translocation of p-FAK and p-Paxillin to peripheral actin cytoskeleton. Cdc42, p-FAK, and p-Paxillin may be involved in SLIT2-mediated suppression of migration [83]. Subsequently, in ESCC, Jiang et al. found miR-1179 promotes cell invasion through SLIT2/ROBO1 axis. Down-regulation of miR-1179 suppressed cell invasion *in vitro* with an increasing level of SLIT2 and ROBO1. Besides, the upregulation of SLIT2 decreased cell invasiveness through ROBO1 [84].

### Pancreatic cancer

Unbiased analysis of the transcriptional network governing the angiogenic switch in human pancreatic cancer identified ROBO1 and SLIT1 as putative proangiogenic genes [85]. In pancreatic cancer, Fu et al. found the miRNA-218 and Robo1 signaling axis may contribute to the lymphatic metastasis of pancreatic cancer. They also first demonstrated downregulation of miRNA-218 and upregulation of ROBO1 in pancreatic cancer [86]. Furthermore, Tang et al. found that the Slit-Robo signaling genes were frequently altered in pancreatic cancer [60]. Subsequently, SLIT2 was found to inhibit leukocyte migration in the gradient of monocyte chemotactic protein-1 (MCP-1) in an *in vitro* pancreatic cancer model [87].

### Intestinal cancer

In intestinal tumorigenesis, Zhang et al. employed several complementary mouse models to clarify the oncogenic function in intestinal tumorigenesis. They showed that SLIT2 and ROBO1 are overexpressed in intestinal tumors. SLIT2-ROBO1 signaling promoted tumorigenesis and tumor growth and this was mediated in part through activation of the Src signaling, which then down-regulated E-cadherin, thereby activation Wnt/β-catenin signaling [88]. Thus, SLIT2-ROBO1 signaling was proposed to play oncogenic role in intestinal tumorigenesis.

## The Slit-Robo signaling pathway in some other solid tumors

In addition, several SLITs and ROBOs are aberrantly expressed during the development of breast [89–91], lung [10, 92], ovarian [93–95], cervical [96] and prostate cancer [97]. In this review, we also briefly summarized the aberrant Slit-Robo pathway in breast and lung tumorigenesis.

### The Slit-Robo signaling pathway in breast cancer

In breast cancer, Slit-Robo has antitumor activity. Most breast tumors have low expression of Slit-Robo and its higher expression is correlated with increased survival rate in cancer patients, whereas low SLIT2 expression is associated with poor survival and increased metastasis [89, 90, 98]. Ectopic SLIT2 expression in breast cancer cells inhibits tumor cell migration and tumor growth in engrafted mice models through a mechanism implicating β-catenin modulation [51]. In breast cancer, SLIT2 blocks a whole host of SDF1-induced signaling involved in motility such as the activation MAP kinase or focal adhesion components [50]. Similarly, overexpression of Slit-Robo in breast cancer leads to down-regulation of CXCR4 and suppression of tumour growth [90]. Recently, Chang et al. demonstrated that activation of Slit-Robo inhibits activation of β-catenin by inhibiting AKT, thereby preventing translocation of cytosolic β-catenin to the nucleus of the fibroblast cells [99]. Furthermore, in breast cancer cells, SLIT2 also inhibits tumor cell migration by affecting the direction of migration through the deubiquitylating enzyme USP33 [100].

### The Slit-Robo signaling pathway in lung cancer

In lung cancer, the expression of SLIT2 is suppressed [10, 92, 101]. Suppression of SLIT2 was associated with a poor patient survival in late-stage diseases [102]. Stimulating SLIT2 expression increased the level of E-cadherin caused by attenuation of its transcriptional repressor SNAI1. Conversely, knocking down SLIT2 expression increased cell migration and reduced cell adhesion through coordinated deregulation of β-catenin and E-cadherin/SNAI1 in the AKT/GSK3β/βTrCP pathway [102]. Furthermore, USP33 was identified to regulates SLIT signaling by stabilizing ROBO1 and was required for SLIT inhibition of lung cancer cell migration [103]. These results suggested that SLIT2 suppresses lung cancer progression.

## Conclusions

In conclusion, the literatures concerning Slit-Robo signaling functions in tumorigenesis are controversial (summarized in Table 1). In most of the cancer types, Slit-Robo acts as a tumor suppressor by inhibiting cell invasion and migration [12, 50, 92, 104–107], except in some gastrointestinal cancers [78].

**Table 1:** Summary of expression and function of SLIT1, SLIT2, SLIT3, ROBO1, ROBO2 and ROBO4 in cancers

Gene	Expression in cancers		Function	Metastasis	References
SLIT1	Upregulation	Prostate cancer	Increased expression in prostate tumors		[97]
SLIT2	Downregulation	Colorectal cancer	Tumour suppress	Suppress	[10, 79, 80]
		Esophageal squamous cell carcinoma	Correlated with poor overall survival	Suppress	[84]
		Intestinal tumors	Promotes intestinal tumorigenesis		[88]
		Breast cancer	Tumour suppressor and low expression is associated with poor survival and increased metastasis	Suppress	[89, 90, 98] [51]
		Lung cancer	Tumour suppressor	Suppress	[10, 92, 101] [102]
		Advanced gastric cancer	Knockdown of SLIT2 in gastric cancer cells promotes cell growth and metastasis *in vitro* and *in vivo*	Suppress	[66, 67]
	Upregulation	Colorectal cancer	Induce malignant transformation of colorectal epithelial cell and tumor metastasis	Promote	[78] [62]
SLIT3	Downregulation	Colorectal cancer	SLIT3 is frequently methylated in colorectal cancers		[10, 77]
		Breast cancer	SLIT3 methylation in breast tumours correlated with reduced SLIT3 expression		[10]
		Liver cancer	SLIT3 was significantly down-regulated in cell lines with high-AFP background		[75]
ROBO1	Downregulation	Breast cancer	A high frequency of allele loss		[18] [69]
		Lung cancer	A high frequency of allele loss		[69]
		Prostate cancer	ROBO1 expression was significantly reduced in prostate tumors		[97]
	Upregulation	Gastric cancer	SLIT2 interacted with ROBO1 to promote gastric cancer invasion	Promote	[62, 64] [63]
		Liver cancer	Overexpressed in and shed into serum for prediction of tumor stage and differentiation status		[76] [75]
		Intestinal tumors	Promotes intestinal tumorigenesis	Promote	[88]
		Pancreatic cancer	Contribute to the lymphatic metastasis of pancreatic cancer	Promote	[86]
		Colorectal cancer	ROBO1 was significantly associated with an increased metastatic risk and poorer overall survival in colorectal carcinoma patients	Promote	[77]
ROBO2	Upregulation	Liver cancer	ROBO2 were significantly up-regulated cell lines with high-AFP background		[75]
ROBO4	Downregulation	Liver cancer	ROBO4 could discriminate poorly differentiated HCC from other subgroups		[75]

However several issues should be addressed in the following study of Slit-Robo pathway in GC. First, the key members in SLIT family and ROBO family which are involved in GC should be identified including expression and mutation status. Due to the detailed mechanism of Slit-Robo signaling exerting its function in gastric tumorigenesis is not clearly and fully understood. Thus the cytoskeleton changes, cell migration and invasion, angiogenesis, proliferation and apoptosis, crosstalk pathways linking to Slit-Robo signaling need to be elucidated. Second, although a hypothetical signal transduction has been proposed in Slit-Robo signaling, we still need to comprehensively investigate how this pathway is regulated. The most important issue is there are still no targetable molecules which can efficiently target the Slit-Robo pathway. Therefore, vast possibilities exist for carrying out additional basic research in this direction to development efficient targeted anticancer therapies.

## Abbreviations

GC: Gastric cancer
ROBO: Roundabout
*H.pylori*: *Helicobacter pylori*
LRR: Leucine-rich repeats
Ig: Immunoglobulin-like
FNIII: Fibronectin type 3
CC: Conserved cytoplasmic
HS: Heparin sulfate
CNS: Central nervous system
GAPs: GTPase-activating proteins
GEFs: Guanine nucleotide-exchange factors
HGF: Hepatocyte growth factor
SDF-1: Stromal derived factor-1
srGAP1: Slit-Robo GTPase activating protein1
IHC: Immunohistochemistry
CRC: Colorectal cancer
HCC: Hepatocellular carcinoma
HGF: Hepatocyte growth factor
EMT: Epithelium mesenchymal transition
ESCC: Esophageal squamous cell carcinoma
MCP-1: Monocyte chemotactic protein-1
